# Upstream Policy Changes to Improve Population Health and Health Equity: A Priority Agenda

**DOI:** 10.1111/1468-0009.12640

**Published:** 2023-04-25

**Authors:** RASHAWN RAY, PAULA M. LANTZ, DAVID WILLIAMS

**Affiliations:** ^1^ University of Maryland; ^2^ University of Michigan; ^3^ Harvard University

**Keywords:** health equity, policy, population health, race, racism

## Abstract

**Policy Points**

- Upstream factors—social structures/systems, cultural factors, and public policy—are primary forces that drive downstream patterns and inequities in health that are observed across race and locations.
- A public policy agenda that aims to address inequities related to the well‐being of children, creation and perpetuation of residential segregation, and racial segregation can address upstream factors.
- Past successes and failures provide a blueprint for addressing upstream health issues and inhibit health equity.

## Introduction

The upstream/downstream metaphor for understanding the root causes or fundamental upstream drivers of population health and how they produce downstream effects, consequences, and inequities is well understood in research, teaching, and public health practice circles. The upstream/downstream framework is visible in the World Health Organization's (WHO's) multilevel conceptual model of the social determinants of health and health inequities that posits that health and well‐being are primarily determined by *upstream social structural factors*. These factors include socioeconomic, cultural, political, and public policy contexts that influence individuals’ socioeconomic position and experiences, as well as how racism and discrimination operate and function within the social structures.^1^ In turn, these macrostructural, or social structural, factors influence a broad set of intermediary social determinants of health at the mesolevels and microlevels (i.e., downstream levels), including what the WHO model refers to as the material conditions of living (housing, food, safety, etc.), health‐related behaviors, biological factors, psychosocial processes, and personal health care services.

All of the factors in the WHO model work in multiple and sometimes bidirectional ways to influence both the expression of social needs and health at the individual level. Nonetheless, it is the upstream factors—social structures/systems, cultural factors, and public policy— that are the primary driving forces behind the stark downstream patterns and inequities in health that we observe across socioeconomic, racial, ethnic, gender, and place lines.^2^ As Williams and Sternthal have articulated: “[s]ocial structure refers to enduring patterns of social life that shape an individual's attitudes and beliefs; behaviors and actions; and material and psychological resources.”^3^

Hurricane Katrina in 2005, as just one example, was an environmental disaster that exposed the fact that the effects of “natural” disasters are not random, nor do they impact populations equally.^4^ The upstream drivers of racial and socioeconomic inequality were the root causes of the differential immediate and long‐term effects of Katrina on people and communities. Predominately Black neighborhoods in New Orleans were located in the “bottoms” and lacked the infrastructure and public policy foundations for adequate housing, transportation, and income security, thus Black people in New Orleans were more likely to die and be displaced.^5^ Hurricane Katrina exposed the role of poverty, racial segregation, and racial discrimination in shaping long‐term socioeconomic health outcomes.^6^

The impact of Hurricane Katrina was less about the actual hurricane and more about upstream social determinants of health that structure deleterious outcomes for people living in marginalized communities. Stewart and Ray used Hurricane Katrina to conceptualize an allegory that encompasses downstream and upstream social determinants of health.^7^ They asked readers to imagine swimming in a body of water and noticing someone drowning. You then swim over to save this person. On reaching the shoreline, you see someone else drowning. You save that person. You then look in the water and see other people drowning as well. The reader is asked to gaze upstream. They see some people at the top of a waterfall in calmer, safer waters, whereas there are others at the bottom of the waterfall having water dumped on them in trepid conditions. The reader is forced to reckon with whether someone or something is actually pushing all of these people in the water to drown, or at least being negligent enough to not stop it.^7^ This upstream metaphor speaks to public policy reform regarding the structural drivers of health inequality, with the waterfall indeed operating as those systemic drivers.

The COVID‐19 pandemic provides another powerful example for how upstream policies, or lack thereof, impact population health. Shortly before the COVID‐19 pandemic, the Global Health Security Index released its inaugural report about pandemic preparedness. Although the United States received the highest overall score (83.5 out of 100), some indicators were concerning and are related to upstream structural issues. The United States scored low on emergency response operations, health capacity, and health care access. A few months later, these low scores were on full display, as the United States was one of the countries hit the hardest by COVID‐19.^8^ Furthermore, in the third year of the pandemic, little has changed. Although the United States still had the highest overall country score (79.4), it ranked 183^rd^ in health care access, 46^th^ in risk communication, and 9^th^ in socioeconomic resistance out of 195 countries.^9^ As Alberti, Lantz, and Wilkins have stated, “The reality is that the United States is ill equipped to realize health equity in prevention and control efforts for any type of health outcome, including an infectious disease pandemic.”^10^ This is important considering research found that health care expenditures and the health care workforce did little to impact the COVID‐19 mortality rate across countries.^11^

The COVID‐19 pandemic further revealed the deep cracks and weaknesses in the United States public health system.^12^ A plethora of studies reveal the differential effects of COVID‐19 on health and mortality across social class and race lines, including that frontline services workers—with increased exposure, no sick leave, and no health insurance—were disproportionately more likely to experience sickness and death during the pandemic.^13^ As with every other type of population health shock, the most socially‐marginalized communities are the most vulnerable.^14^

## The Role of Public Policy

Public policy plays a quintessential role in shaping the myriad of upstream macro and structural forces that cascade downstream to create both social and health inequities in the communities that comprise populations. In turn, this means that addressing the fundamental root causes of population health problems and inequities must involve significant redirection and reform of the public policies that shape our social structures, systems, and institutions.

Although the list of public policies (both individual policies and bundled or linked policy systems) that are in need of significant reform to improve population health is long and complex, in this paper, we present our top priority areas for serious attention and change. We assert that three primary upstream public policy drivers of social and health inequality must be addressed to improve overall population health and to reduce health inequities in the United States:

1. Public policy related to the well‐being of children (e.g., reducing poverty, establishing income security, and creating high‐quality pre‐K).
2. Public policy related to the creation, perpetuation, and legacies of residential segregation with a particular focus on housing affordability and addressing the devaluation of property of predominately Black neighborhoods that helps drive the racial wealth gap.
3. Public policy related to reducing racial discrimination (both structural and interpersonal) related to key social determinants of health.

We acknowledge that some recent upstream gains (before the COVID‐19 pandemic) deserve note, an overwhelming majority of which were driven by public policy initiatives and reforms at the national and state levels. First, in regard to some key socioeconomic indicators, rates of high school graduation and college enrollment have been trending upward in the United States across all racial and ethnic groups, although higher education degree completion trajectories and educational debt burdens remain significantly different.^15^

Second, for the purposes of discussing upstream drivers of health, the Affordable Care Act (ACA) actually broadened the civil rights landscape in which the health care system and insurance industry operate.^16^ The ACA also increased health insurance coverage for the most marginalized Americans and helped to increase the use of clinical preventive services, improve access to care for acute and chronic conditions, and expand the number and reach of federally qualified health centers.^17^, ^18^ Buchmueller and colleagues found that the ACA significantly decreased uninsurance rates among Black, Latino, and White populations; among Black people, the uninsured population decreased roughly 20% in the first six years.^19^ Improvements in insurance coverage and overall health were especially pronounced in states that expanded Medicaid, including evidence of reduced mortality in these states.^20^

Third, it should be acknowledged that life expectancy in the United States was steadily increasing until the impact of the drug overdose epidemic, and then the COVID‐19 pandemic hit with great force. From 1990 to 2018, life expectancy increased nearly 6 years for Black people. Nonetheless, life expectancy for Black and Indigenous populations still lag far behind that of White and Latino populations.^21^ Even beyond health care coverage and gaps in care, upstream problems contribute substantially to racial gaps in life expectancy. As noted above, upstream problems have been on full display during COVID‐19. Although life expectancy decreased for all groups, Black Americans and Indigenous populations faced the most severe decreases during the COVID‐19 pandemic.

## Public Policy Reforms Focused on the Well‐Being of Children

A large and growing body of evidence demonstrates the importance of addressing issues linked to childhood poverty for health and well‐being across the life course.^22^ Research reveals that poor children, compared with children of a higher socioeconomic status, are exposed to more family turmoil, violence, separation, instability, and chaotic households. They also experience less social support and have parents that are less responsive and more authoritarian. Research shows that children from lower socioeconomic statuses read less frequently, watch more TV, have less access to books and computers, and are less likely to have parents involved in their school activities. In addition, poor children are more likely to consume air and water that is polluted; reside in homes that are more crowded, noisier, and of lower quality; live in neighborhoods that are more dangerous; have poorer city services; and have greater physical deterioration. They are also more likely than their economically advantaged peers to attend schools and day care that are of an inferior quality.^23^

Childhood poverty rates—although still shamefully high in the United States—have been falling since a peak rate of 23% in 2012 and reach a record low in 2019.^24^ A report by Child Trends shows that the past three decades experienced the most significant drop in child poverty rates.^25^ In fact, child poverty fell 59% from 1993 to 2019. This decrease is attributed to key public policies, such as the Earned Income Tax Credit and higher minimum wages in states as well as low unemployment rates among single mothers. Although child poverty decreased, racial gaps persist among low‐income families. President Biden's Infrastructure Investment and Jobs Act (2021) and Inflation Reduction Act (2022) have the potential to contribute greatly to addressing the well‐being of children. In this issue, Pilkauskas goes into further detail about how income support can serve as a policy solution to address upstream health challenges.^26^

Research also reveals that intensive, high‐quality early childhood intervention programs can have large, positive, long‐term, educational, and physical and mental health impacts on children from disadvantaged backgrounds. The Perry Preschool Program was a 2‐year intervention in which Black children ages 3 to 4 years, living in poverty, were randomized to participate in a preschool program or to be in a control group. The program consisted of daily morning classes at school, and weekly afternoon home visits from the teacher.^27^ At age 40, compared with the control group, those who received the program were more likely to have graduated from high school and college. They also had higher levels of employment, income, health insurance, savings, and home ownership, and lower rates of arrests (for violent, property, and drug crimes), out‐of‐wedlock births, and welfare assistance.^28^ Moreover, positive effects were also evident for siblings (especially male siblings) of participants in the program.

Striking additional evidence for the long‐term benefits of early childhood interventions comes from the Abecedarian program, an experimental study of early child education that randomized poor children (80% of whom were Black) at birth and provided an intensive program from birth to 5 years old and has followed them into adulthood. The program offered a safe and nurturing environment, good nutrition, intellectual stimulation, and pediatric care. At age 21, individuals in the intervention condition had fewer depressive symptoms, lower marijuana use, a more active lifestyle, better academic performance, and better vocational success than those in the control group.^29^, ^30^ By their mid‐30s, the intervention group members had lower levels of risk factors for cardiovascular and metabolic disease (such as high blood pressure and obesity), with the positive effects being stronger for males than for females.^31^

There is growing evidence that addressing childhood poverty by providing additional income to parents then enhances family economic security and is causally linked to improvements in a broad and diverse range of child and youth outcome topics.^32^ Studies using a range of research designs document the benefits to parents and children of interventions that enhance economic security for expectant parents and parents with children. The income enhancement policies studied have ranged from minimum wage laws to Federal Earned Income Tax Credit and other reforms to tax policy.^31^ A report from the National Academy of Sciences in 2019 outlined an ambitious agenda that indicates multiple options for the United States to reduce child poverty by 50% within a decade. These policy options include combinations of Earned Income Tax Credits, the expansion of housing vouchers, the Supplemental Nutrition Assistance Program, child allowances, child and dependent care tax credits, work‐based programs, government and tax transfers, and public health insurance.

An innovative program called Baby Bonds, championed by economist Darrick Hamilton, is receiving considerable policy interest at the state and local levels in recent years.^33^ For example, the state of Connecticut and the District of Columbia have implemented a plan that would give each poor (Medicaid‐eligible) baby a trust fund of $3,200 that would be established and guaranteed by the government, which should grow to >$10,000 by the time the child turns 18 years old. The state of Connecticut estimates that this bond program will enroll about 16,000 children annually, so that disadvantaged 18‐year‐olds will have resources to narrow the gap between themselves and their wealthier peers. This money could be used for education, purchasing a home, or other needs. The policy is officially race‐neutral but would give a major, new source of financial assistance to racial and ethnic, low‐income groups.

## Public Policy Reforms that Address the Ongoing Legacies of Residential Segregation

The built and natural environments are evaluated based on their accessibility and quality of public space coupled with existing or changing social environments (e.g., segregation and gentrification).^33^ The structural components of the built and natural environments that are used for physical activity and public transportation are often less available to communities of color. Collectively, these challenges provide a clearer understanding of why people living in communities are more likely to be obese and diagnosed with high blood pressure and be exposed to gun and police violence.^34^ Accordingly, the physical design of communities can exacerbate race‐based health disparities,^35^ and the constraints of these structural components further expose communities of color to gun and police violence through mechanisms of hypervisibility and racial profiling.

Black neighborhoods are more likely to be situated near toxic waste sites and pollution‐producing facilities. Consequently, Black communities are much less likely to have clean water and air. In turn, Black children are more likely to be diagnosed with asthma and other health disorders. We simply have to look at the Flint, Michigan crisis that started in 2014 when a governmental decision to switch the city's municipal water source dramatically increased the leeching of lead from older water pipes and in turn dramatically increased both lead poisoning in children and Legionnaires’ disease. These deleterious health outcomes, however, are not just in Flint. In Baltimore (where the Black population over 60%), lead levels in children are over double the recommended rate. Lead exposure not only causes physical health issues, but it harms cognitive development, rational decision‐making, and academic test scores. The United States pays about $15 billion annually to deal with lead poisoning cases. Jackson, Mississippi is the most recent predominately Black city to experience a catastrophe related to the basic public good of water. Jackson's water crisis is so severe that schools were forced to close. Imagine if lead problems did not exist in places like Baltimore, Flint, Jackson, and hundreds of other communities nationwide and instead, those funds could be used to improve schools, neighborhood infrastructure, and health resources.^36^

Inefficiencies in the social service infrastructure frequently compounds health issues for low‐income Americans. The focus on acute social service needs downstream detracts attention and funding from the upstream root causes of people drowning at the bottom of the waterfall. For example, places that lack an efficient social service infrastructure also have serious problems with housing affordability and instability. Housing and social services are fundamentally important because they are supposed to protect the most vulnerable population: children.^37^ In this special issue, Medipanah notes that affordable housing as well as homeownership rates are key upstream drivers of health inequality.^38^

Collectively, these issues speak to environmental challenges driven by racial residential segregation. Policies that ensure cleaner energy, air, and water will lead to healthier communities, particularly for communities of color. We mentioned President Biden's recent legislation above. Creating cleaner air, on one hand, and addressing the historical legacies of highways in Black community, on the other hand, actually go hand‐in‐hand and serve as upstream drivers of health inequality.

Once affordable housing is addressed, the racial gap in homeownership and the devaluing of predominately Black neighborhoods must be rectified. In an analysis of cities across the United States, Perry found that, on average, homes in predominately Black neighborhoods are valued at $48,000 less than homes in predominately White neighborhoods.^39^ Bank of America's new program to close the housing and racial wealth gaps uses on‐time payments for utilities and does not require a down payment for new homeowners who live in select, historically redlined cities. This is a policy idea that policy experts have recommended. Instead of using a flawed credit system that has racism baked within it, we suggest using actual utility bills to showcase credit worthiness.^40^ In addition to the banking industry, which has a long and torrid history of discriminating against Black people in gaining access to home loans, state and federal governments could also build on this program to provide more equitable access to homeownership.

## Public Policy Reforms to Reduce Interpersonal and Structural Racism in Key Social Systems/Institutions

Research shows the diminishing returns that racial/ethnic populations receive from their socioeconomic status.^41^, ^42^ This research illuminates the ways that inequities in health care operate across and within socioeconomic status and social contexts. As Brown and Hohman as well as Michener and Ford highlight in this special issue, addressing systemic oppression is paramount to improving health outcomes.^43^, ^44^

Accordingly, downstream policies that do not address how structural racism contributions to upstream health inequities will fail to have the impacts they should.^45^ It is important to discuss how gun violence, policing, and the criminal justice system contribute to upstream health problems. For young males, particularly Black males, gun violence is a leading cause of death.

Lack of a social services and health infrastructure expose people to community risks, such as gun violence.^46^ Homicide, mostly due to gun violence, is one of the top causes of death for Black men.^47^ For Black men under 45 years of age, homicide is the number one cause of death. However, often not framed in a similar way, homicide is also a top five cause of death for White men under the age of 45. The same goes for Latino men too. It is clear that gun violence, and violence more broadly among young males, is a substantial problem. Gun violence also has upstream consequences that impact the response of first responders and treatment once in medical facilities. Gun violence also exposes the community to a higher prevalence of mental health issues.^48^

In addition to the traditional ways we think about gun violence, police violence plays a prominent role in community health. Data from the Surveillance for Violent Deaths National Violent Death Reporting System suggest that police killings are the third leading cause of violence‐related deaths accounting for nearly 25% of the >16,000 violence‐related deaths in 16 states.^49^ In fact, research states that police officers are just as likely to kill Black people with a high income as they are to kill Black people with a low income. Police officers are 3.5 times more likely to kill Black people who are unarmed and not attacking compared to White people who are unarmed and not attacking. Police officers are 21 times more likely to kill Black teenagers than they are to kill White teenagers.^49^

In addition to the obvious health impacts (e.g., death, injury) of overpolicing, police killings influence the health profiles of local communities. Research shows that aggressive policing leads to worse mental and physical health for people living in overpoliced communities.^50^, ^51^ For example, aggressive and excessive police tactics debilitate health, increase symptoms of trauma and anxiety, worsen self‐rated health, and provoke clinical levels of psychological distress.^52^ Moreover, people living in communities that are more exposed to police violence report a whole host of health conditions, including poorer self‐rated health, higher levels of diabetes, higher blood pressure, asthma episodes, obesity, and psychological distress.^53^, ^54^

A series of policy changes has been advanced to address gun violence and police violence. However, they are actually different social processes. An analysis by Mapping Police Violence documents that cities with the highest levels of police violence are often not the same cities with the highest level of violent crime. Accordingly, it is important to decouple police violence from violent crime and realize they are two distinct social problems that need to be addressed separately. Accordingly, addressing gun violence will require policies that not only regulate guns, but deal with the ghost guns that run rapid in predominately Black, low‐income communities. It also means creating a better social service infrastructure that helps provide educational and work opportunities for marginalized youth. To address police violence, policies must increase the accountability of law enforcement. Ray has advanced the importance of creating police department and police officer liability insurance to shift financial liability from taxpayers to police.^55^ Colorado has advanced this policy and other cities and states are considering various configurations.

## Conclusion

“Of all the forms of inequality, injustice in health care is the most shocking and inhumane.” Dr Martin Luther King Jr made this comment in 1966 at the Chicago Press Conference in connection with the Medical Committee for Human Rights meeting. King lamented that despite pursuits to address racial segregation and poverty, health and health care inequality seemed to materialize in ways that manifest across race, gender, and social class lines. Well over 50 years later, his comments continue to ring true. The research is clear on the advancement of both health care and social policy reforms that will bend the arc of justice toward a more equitable and healthier society.

In this article, we have aimed to layout a series of policy‐focused strategies and practices to address upstream determinants of health to establish humanity for everyone. Our article highlights past gains and successes as well as failures and continuing problems that contribute to upstream health issues and inhibit health equity. Granted, what we unpacked here is not an exhaustive list. Rather, we aimed to highlight the most persistent and impact upstream factors that can be addressed through public and social policy reform and innovation.

Given the extremely divisive nature of the current political landscape in the United States, one might ask what, if anything, in this agenda is politically feasible? This question reflects a conservative, reservist, and reactionary perspective. It is clear that civil and human rights are being rolled back in certain states and also at the federal level, including the June 2022 Supreme Court ruling that overturned Roe v. Wade.^56^ In addition, the growing efforts to prohibit the teaching of critical race theory, structural racism, and other “divisive constructs” at the local and state levels represent intense pushback against the recognition that institutions, systems, and social structures—including public policy—can embody and perpetuate racism.^57^

If the goal is improved health of the communities and all people in the United States, then the actual policy agenda needed to achieve that goal has to be clear, bold, and well‐reasoned. Although we understand the enormous challenges of progressive policy reform at the present time, we remain committed to the use of valid and reliable evidence and high‐quality research to provide a pathway forward that is beneficial to Americans at the aggregate level rather than engaging in political fissures.

*Funding/Support*: This study was funded by the Milbank Foundation.

*Conflict of Interest Disclosures*: None declared.
